# Characterization of Benign Breast Diseases and Association With Age, Hormonal Factors, and Family History of Breast Cancer Among Women in Sweden

**DOI:** 10.1001/jamanetworkopen.2021.14716

**Published:** 2021-06-25

**Authors:** Annelie Johansson, Athanasia E. Christakou, Adina Iftimi, Mikael Eriksson, Jose Tapia, Lambert Skoog, Christopher C. Benz, Kenny A. Rodriguez-Wallberg, Per Hall, Kamila Czene, Linda S. Lindström

**Affiliations:** 1 Department of Oncology and Pathology, Karolinska Institutet and University Hospital, Stockholm, Sweden; 2 Department of Biosciences and Nutrition, Karolinska Institutet, Stockholm, Sweden; 3 Department of Medical Epidemiology and Biostatistics, Karolinska Institutet, Stockholm, Sweden; 4 Department of Medicine, University of California, San Francisco; 5 Buck Institute for Research on Aging, Novato, California; 6 Division of Gynecology and Reproduction, Department of Reproductive Medicine, Karolinska University Hospital, Stockholm, Sweden; 7 Department of Oncology, Södersjukhuset, Stockholm, Sweden

## Abstract

**Importance:**

Benign breast diseases (BBDs) are common and associated with breast cancer risk, yet the etiology and risk of BBDs have not been extensively studied.

**Objective:**

To investigate the risk of BBDs by age, hormonal factors, and family history of breast cancer.

**Design, Setting, and Participants:**

This retrospective cohort study assessed 70 877 women from the population-based Karolinska Mammography Project for Risk Prediction of Breast Cancer (KARMA) who attended mammographic screening or underwent clinical mammography from January 1, 2011, to March 31, 2013, at 4 Swedish hospitals. Participants took part in a comprehensive questionnaire on recruitment. All participants had complete follow-up through high-quality Swedish national registers until December 31, 2015. Pathology medical records on breast biopsies were obtained for the participants, and BBD subtypes were classified according to the latest European guidelines. Analyses were conducted from January 1 to July 31, 2020.

**Exposures:**

Hormonal risk factors and family history of breast cancer.

**Main Outcomes and Measures:**

For each BBD subtype, incidence rates (events per 100 000 person-years) and multivariable Cox proportional hazards ratios (HRs) with time-varying covariates were estimated between the ages of 25 and 69 years.

**Results:**

A total of 61 617 women within the mammographic screening age of 40 to 69 years (median age, 53 years) at recruitment with available questionnaire data were included in the study. Incidence rates and risk estimates varied by age and BBD subtype. At premenopausal ages, nulliparity (compared with parity ≥3) was associated with reduced risk of epithelial proliferation without atypia (EP; HR, 0.62; 95% CI, 0.46-0.85) but increased risk of cysts (HR, 1.38; 95% CI, 1.03-1.85). Current and long (≥8 years) oral contraceptive use was associated with reduced premenopausal risk of fibroadenoma (HR, 0.65; 95% CI, 0.47-0.90), whereas hormone replacement therapy was associated with increased postmenopausal risks of epithelial proliferation with atypia (EPA; HR, 1.81; 95% CI, 1.07-3.07), fibrocystic changes (HR, 1.60; 95% CI, 1.03-2.48), and cysts (HR, 1.98; 95% CI, 1.40-2.81). Furthermore, predominantly at premenopausal ages, obesity was associated with reduced risk of several BBDs (eg, EPA: HR, 0.31; 95% CI, 0.17-0.56), whereas family history of breast cancer was associated with increased risk (eg, EPA: HR, 2.11; 95% CI, 1.48-3.00).

**Conclusions and Relevance:**

These results suggest that the risk of BBDs varies by subtype, hormonal factors, and family history of breast cancer and is influenced by age. Better understanding of BBDs is important to improve the understanding of benign and malignant breast diseases.

## Key Points

**Question:**

What is the risk of benign breast diseases (BBDs) and how are they associated with age, hormonal breast cancer risk factors, and family history of breast cancer?

**Findings:**

In this cohort study of 61 617 women in Sweden, incidence rates for distinct BBD subtypes in individuals 25 to 69 years of age were estimated. The study found that the risk of the most common BBDs by hormonal factors was influenced by age and that family history of breast cancer was associated with the risk of proliferative and nonproliferative BBDs.

**Meaning:**

These results indicate that age and hormonal factors influence the risk of BBDs and that a better understanding of BBDs is important to improve our understanding of breast diseases as a whole.

## Teaser

This cohort study investigates the risk of benign breast diseases in women by age, hormonal breast cancer risk factors, and family history of breast cancer.

## Introduction

Benign breast diseases (BBDs) are common throughout a woman’s lifetime, from early reproductive life to the postmenopausal part of life, making it a potential health concern to a large number of women.^1,2,3,4,5,6^ The etiology and risk of BBDs have not been extensively studied, despite an increasing incidence of BBDs detected by population-based mammographic screening.^7,8^ However, the exact incidence rates are unexplored. Moreover, previous studies^1,2,9^ on BBDs have often been small, inconsistent in their pathological disease classification, and conducted before mammographic screening programs were introduced. Consequently, to our knowledge, there are no up-to-date studies of the incidences of distinct BBD subtypes, and risk factors have not been well established. Furthermore, given that the risk of BBD diagnosis extends throughout several decades in a woman’s life, it is important to understand how age influences the risk.

Hormonal factors (ie, reproductive and lifestyle factors that affect hormonal exposure, including exogenous hormone use) are well known to affect a woman’s breast cancer risk, but little is known regarding hormonal factors and BBDs. Factors such as early menarche, regular and short menstrual cycles, nulliparity, older age at first birth, use of oral contraceptives and hormone replacement therapy (HRT), and high postmenopausal body mass index (BMI) are associated with a higher breast cancer risk, whereas longer breastfeeding duration and higher premenopausal BMI are associated with a reduced risk.^10,11,12,13^ Of importance, a history of certain BBDs is also a risk factor for breast cancer; benign proliferative disease with or without atypia increases the risk approximately 4- and 2-fold, respectively, whereas it is less clear whether nonproliferative diseases affect the risk.^7,9,14,15,16,17^ In addition, family history of breast cancer influences the risk of breast cancer after a BBD diagnosis, and the risk may be further elevated in younger women.^7,14,15^ Therefore, it is important to improve our understanding of benign vs malignant breast diseases for breast cancer risk assessment.

A previous study^14^ categorized BBDs according to their association with breast cancer risk. However, more detailed classification of the diseases is needed in accordance with recent pathological guidelines.^18^ Indeed, BBDs cover a group of various noninvasive breast conditions, including but not limited to, epithelial proliferation with atypia (EPA) or epithelial proliferation without atypia (EP) (hyperplasia), fibroadenomas (firm breast masses that often affect younger women), papillomas (small discrete benign tumors), adenosis (enlargement of the lobules), calcifications, fluid-filled cysts, and fibrocystic changes (FCCs) (composed of cysts and solid lesions).^19,20^

In this study, we aimed to determine the incidence and risk of in-detailed classified BBDs in 67 617 women from the Karolinska Mammography Project for Risk Prediction of Breast Cancer (KARMA) cohort. Information on breast biopsy specimens was extracted from pathology medical records and thoroughly classified into BBD subtypes according to the latest European guidelines. Furthermore, we investigated how hormonal factors and family history of breast cancer are associated with the risk for BBDs, with special interest in how the risk is influenced by age.

## Methods

### Study Participants

KARMA is a population-based and well-characterized Swedish screening cohort.^21^ Between January 1, 2011, and March 31, 2013, women who attended the national mammographic screening program or underwent clinical mammography at 4 Swedish hospitals located in Stockholm (Södersjukhuset) and Skåne County (Helsingborg, Lund, and Landskrona) were invited to participate. In 2011, all women 40 to 69 years of age were invited for screening, which was extended to those 70 to 74 years of age in 2012.^22^ A total of 70 877 women were recruited to the cohort.^21^ On recruitment, participants answered a comprehensive web-based questionnaire on reproductive and lifestyle factors. The ethical review board at Karolinska Institutet in Stockholm approved the KARMA study, and the approval applied to use of the data in the current study. All participants signed informed consent forms at recruitment.^21^ All data were deidentified. This study followed the Strengthening the Reporting of Observational Studies in Epidemiology (STROBE) reporting guideline.

Detailed information of the KARMA participants before and after inclusion to the study is available through national and regional registries. All residents in Sweden have a unique personal identification number, which enables linkage to high-quality national registries of high validity and essentially complete coverage, including the Swedish Cancer Register and the Swedish Cause of Death Register.^23,24,25,26^ Furthermore, records on breast biopsies (including cytologic fine-needle aspirations) are retrieved from the regional pathology medical record system SymPathy. Diagnoses in the Swedish Cancer Register and SymPathy are classified by the *International Classification of Diseases for Oncology (ICD-O)*^27^ and Systematized Nomenclature of Medicine.^28^ Registry-based data and information from SymPathy are continuously updated, every 2 years, for the KARMA participants. The current analyses were conducted from January 1 to July 31, 2020.

### BBD Classification

Diagnoses from SymPathy were categorized into 10 BBD subtypes according to the latest European pathology guidelines of breast disease^18,29^ and in consultation with an experienced breast pathologist (L.S.). The following BBD subtypes were included: EPA (ie, atypical hyperplasia), EP (ie, hyperplasia), adenosis (including sclerosing adenosis), papilloma, calcifications, fibroadenoma, FCCs (eg, fibrosis and cystic fibroadenosis), cysts, inflammation (chronic and granulomatous), and nonepithelial tumors (eg, lipoma). For more details, see eMethods and eTable 1 in the Supplement.

### Statistical Analysis

Each BBD subtype was analyzed separately, with the diagnosis of the analyzed BBD subtype as the outcome of interest. When a participant was diagnosed with the same BBD subtype multiple times, only the first diagnosis was considered. Given that the mammographic screening age was 40 to 69 years at the start of KARMA, all women 40 to 69 years of age at recruitment with available questionnaire data were included in the study. Follow-up started retrospectively at the age of 25 years and ended at diagnosis of the analyzed BBD, breast cancer diagnosis, death, or end of follow-up after December 31, 2015 (right censoring). Women who were older than 25 years at the start of SymPathy were followed up from 1979 (left truncation).

The incidence rates for the BBD subtypes were estimated by calculating the events per 100 000 person-years between the ages of 25 and 69 years. The risk of BBDs by hormonal factors and family history of breast cancer (defined as mother or sister diagnosed with the disease) was assessed by Cox proportional hazards regression with age as the time scale, adjusting for KARMA unit, birth cohort, and educational level. Family history of breast cancer was additionally adjusted for number of sisters. The participants were followed up in 3 age groups, 25 to 44 years, 45 to 54 years, and 55 to 69 years, defined as premenopausal, perimenopausal, and postmenopausal ages, respectively. Hormonal factors included reproductive factors (age at menarche, menstrual cycle regularity and length, parity, age at first birth, and breastfeeding duration), BMI (calculated as weight in kilograms divided by height in meters squared), and use of oral contraceptives and HRT. When applicable, the covariates were allowed to change over time (time-varying covariates).^30^ For more details on the covariates, see eMethods in the Supplement. The covariate information was self-reported retrospectively in the questionnaire but analyzed as if it were collected prospectively. This method relies on the assumption that the risk of death is independent on BBD diagnosis not to have a bias in the estimated hazard ratios (HRs).

The HRs and their 95% CIs were summarized in tables and forest plots. *P* < .05 was treated as statistically significant, and all statistical tests were 2-sided. Data preparation and analyses were performed in R statistical software, version 3.5.2 (R Foundation for Statistical Computing). Survival analyses were conducted using the R functions coxph and tmerge in package survival.

## Results

**Figure 1.**
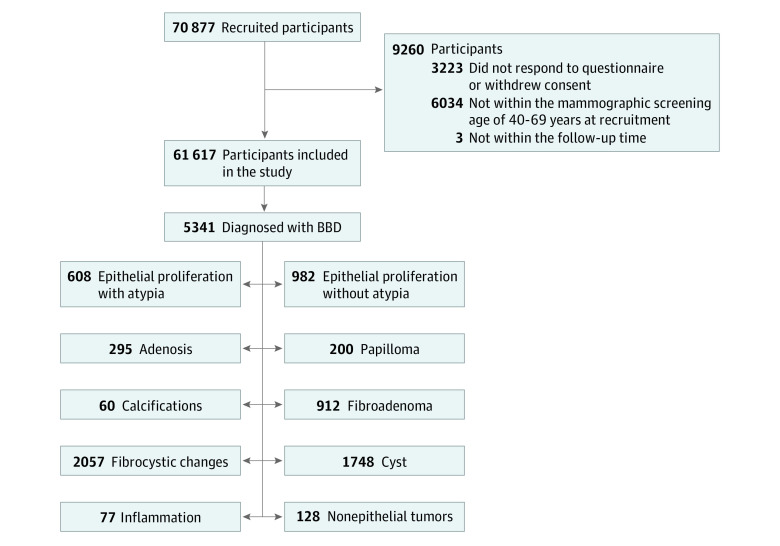
Flow Diagram of the Karolinska Mammography Project for Risk Prediction of Breast Cancer (KARMA) Cohort and Benign Breast Diseases (BBDs)

A total of 61 617 women within the mammographic screening age of 40 to 69 years (median age, 53 years) at recruitment with available questionnaire data were included in the study. Of these, 5341 had been diagnosed with a BBD (Figure 1). Baseline characteristics of all 61 617 KARMA participants included in the study are presented in eTable 2 in the Supplement. A total of 87% of the women reported regular menstrual cycles, and 13% were nulliparous at recruitment. Oral contraceptives had been used by 82% of the participants and HRT by 12% of the participants. Furthermore, 13% had a mother or sister who had been diagnosed with breast cancer. Median follow-up for all analyses was 35 years (range, 1-37 years).

### Age Incidences

**Figure 2.**
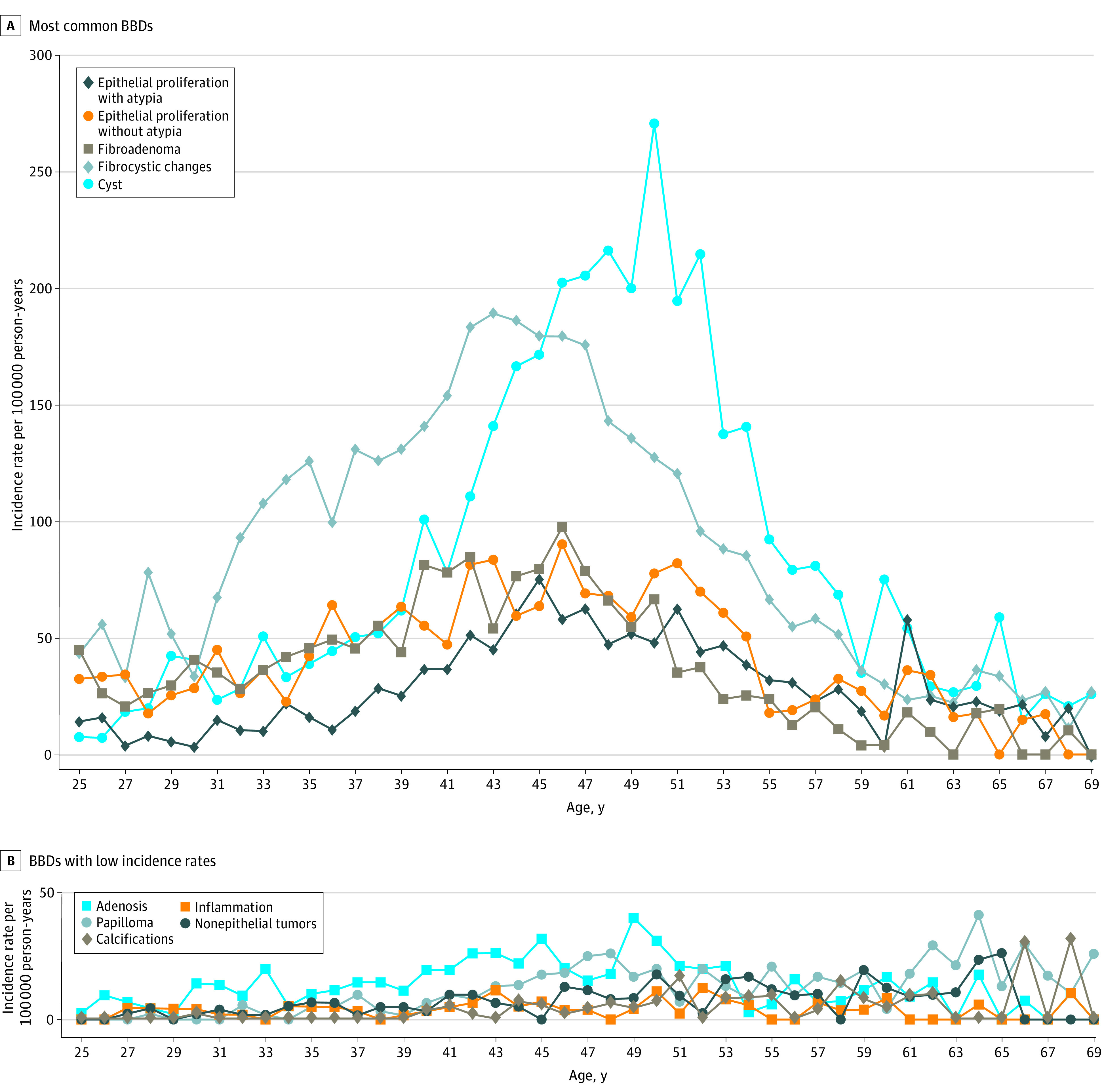
Age Incidences of Benign Breast Diseases (BBDs) in the Karolinska Mammography Project for Risk Prediction of Breast Cancer (KARMA) Cohort Incidence rates (events per 100 000 person-years) were estimated for the BBDs in the KARMA cohort between the ages of 25 and 69 years.

Incidence rates (events per 100 000 person-years) varied by age and BBD subtypes (Figure 2; eTable 3 in the Supplement). Fibroadenoma, EP, and fibrocystic changes (FCCs) were relatively common at younger ages (45, 32, and 42 per 100 000 person-years at the age of 25 years), increased during the 30s and 40s (81, 55, and 140 per 100 000 person-years at the age of 40 years), and decreased thereafter (24, 18, and 65 per 100 000 person-years at the age of 55 years) (Figure 2A). The incidences for FCCs peaked at the age of 43 years (188 per 100 000 person-years) and EP and fibroadenoma at the age of 46 years (90 per 100 000 person-years for EP and 98 per 100 000 person-years for fibroadenoma). Epithelial proliferation with atypia was slightly less common at younger ages, with a maximum at the age of 45 years (76 per 100 000 person-years), and the incidence rates for cysts increased rapidly after 40 years of age, with a maximum at the age of 50 years (271 per 100 000 person-years) (Figure 2A).

The incidence rates for the other BBDs were relatively low with a maximum of 40 for adenosis, 41 for papilloma, 31 for calcification, 12 for inflammation, and 26 for nonepithelial tumors per 100 000 person-years (Figure 2B). Hence, because of otherwise insufficient power, the following analyses focused on EPA, EP, fibroadenoma, FCCs, and cysts.

### Risk of BBDs by Hormonal Factors

#### Age at Menarche and Menstrual Cycle Patterns

Regular menstrual cycles, compared with irregular cycles, were associated with significantly increased risk of fibroadenoma at premenopausal ages (HR, 1.37; 95% CI, 1.02–1.84) (Table 1; eFigure in the Supplement). However, at postmenopausal ages, a history of regular cycles was associated with significantly reduced risk of fibroadenoma (HR, 0.36; 95% CI, 0.17–0.76) but an increased risk of EPA (HR, 8.00; 95% CI, 1.11–57.63). No clear associations between age at menarche (<12 vs >14 years of age) or menstrual cycle length (<27 vs >30 days) and the risk of BBDs were observed for any of the analyzed age groups (Table 1).

**Table 1.** Risk of Benign Breast Diseases by Hormonal Factors

Hormonal factor by age group^a^	HR (95% CI)^b^				
	Epithelial proliferation		Fibroadenoma	Fibrocystic changes	Cyst
	With atypia	Without atypia			
Early (<12 y) vs late (>14 y) age at menarche					
Premenopausal	0.72 (0.43-1.21)	1.33 (0.96-1.84)	0.92 (0.65-1.29)	0.94 (0.75-1.18)	0.94 (0.70-1.26)
Perimenopausal	0.67 (0.39-1.15)	0.61 (0.37-1.02)	0.78 (0.49-1.22)	0.94 (0.69-1.28)	0.90 (0.70-1.16)
Postmenopausal	1.81 (0.80-4.10)	1.23 (0.56-2.69)	1.20 (0.36-3.97)	1.47 (0.76-2.85)	1.55 (0.91-2.64)
Regular vs irregular menstrual cycles					
Premenopausal	0.81 (0.57-1.14)	1.17 (0.88-1.57)	1.37 (1.02-1.84)^c^	1.14 (0.95-1.38)	1.15 (0.90-1.48)
Perimenopausal	1.05 (0.69-1.59)	0.85 (0.60-1.20)	1.27 (0.84-1.94)	0.99 (0.76-1.28)	0.96 (0.78-1.19)
Postmenopausal	8.00 (1.11-57.63)^c^	0.84 (0.38-1.86)	0.36 (0.17-0.76)^c^	0.67 (0.39-1.14)	0.99 (0.58-1.69)
Short (<27 d) vs long (>30 d) menstrual cycle length					
Premenopausal	1.24 (0.75-2.07)	0.82 (0.57-1.17)	0.94 (0.67-1.31)	0.91 (0.72-1.14)	0.90 (0.66-1.22)
Perimenopausal	1.09 (0.63-1.89)	1.33 (0.81-2.16)	0.89 (0.54-1.48)	1.04 (0.77-1.42)	0.86 (0.66-1.11)
Postmenopausal	1.53 (0.64-3.67)	1.48 (0.54-4.04)	0.28 (0.05-1.55)	0.92 (0.47-1.78)	0.85 (0.47-1.54)
Nulliparity vs ≥3 children					
Premenopausal	0.94 (0.60-1.49)	0.62 (0.46-0.85)^c^	1.19 (0.88-1.61)	1.05 (0.85-1.29)	1.38 (1.03-1.85)^c^
Perimenopausal	1.25 (0.79-1.96)	1.01 (0.68-1.50)	1.42 (0.95-2.14)	1.00 (0.75-1.33)	0.89 (0.70-1.13)
Postmenopausal	1.18 (0.51-2.73)	1.41 (0.51-3.87)	0.25 (0.03-2.07)	0.78 (0.38-1.60)	0.76 (0.41-1.39)
Older (>32 y) vs young (<23 y) age at first birth					
Premenopausal	0.62 (0.36-1.06)	0.77 (0.54-1.09)	0.59 (0.40-0.87)^c^	1.04 (0.82-1.31)	1.03 (0.75-1.42)
Perimenopausal	0.82 (0.50-1.35)	0.97 (0.64-1.46)	0.78 (0.46-1.32)	1.23 (0.89-1.72)	1.05 (0.81-1.35)
Postmenopausal	0.67 (0.24-1.86)	1.20 (0.38-3.81)	1.56 (0.50-4.85)	0.84 (0.39-1.78)	0.86 (0.42-1.79)
Did not breastfeed or short (<3 mo) vs longer (>12 mo) breastfeeding duration^d^					
Premenopausal	1.18 (0.72-1.93)	0.81 (0.54-1.20)	1.14 (0.80-1.63)	1.09 (0.88-1.36)	0.96 (0.67-1.37)
Perimenopausal	1.12 (0.71-1.79)	0.98 (0.63-1.53)	0.60 (0.34-1.07)	0.71 (0.52-0.96)^c^	0.87 (0.67-1.13)
Postmenopausal	1.12 (0.46-2.71)	1.09 (0.42-2.86)	0.86 (0.25-2.98)	0.51 (0.23-1.15)	0.79 (0.45-1.38)
Obese (BMI >30.0) vs normal (BMI of 18.5-25.0)					
Premenopausal	0.31 (0.17-0.56)^c^	0.74 (0.55-1.00)	0.56 (0.41-0.78)^c^	0.53 (0.43-0.65)^c^	0.53 (0.40-0.71)^c^
Perimenopausal	0.69 (0.46-1.04)	0.74 (0.51-1.07)	1.05 (0.73-1.50)	0.48 (0.35-0.65)^c^	0.43 (0.33-0.56)^c^
Postmenopausal	0.66 (0.29-1.48)	1.29 (0.64-2.60)	0.57 (0.17-1.90)	0.93 (0.51-1.73)	0.52 (0.30-0.89)^c^

Abbreviations: BMI, body mass index (calculated as weight in kilograms divided by height in meters squared); HR, hazard ratio.

^a^ Premenopausal ages are 25 to 44 years; perimenopausal ages, 45 to 54 years; and postmenopausal ages, 55 to 69 years.

^b^ Cox proportional hazards regression with time-varying covariates using age as the time scale adjusted for Karolinska Mammography Project for Risk Prediction of Breast Cancer unit, birth cohort, and educational level.

^c^ Significant association at *P* < .05.

^d^ Breastfeeding duration analyzed in parous women only.

#### Parity-Related Factors

At premenopausal ages, being nulliparous vs having given birth to 3 or more children was significantly associated with a reduced risk of EP (HR, 0.62; 95% CI, 0.46–0.85) but a significantly increased risk of cysts (HR, 1.38; 95% CI, 1.03–1.85) (Table 1; eFigure in the Supplement). However, compared with being uniparous, being nulliparous was associated with a reduction in the risk of cysts of 26% (HR, 0.74; 95% CI, 0.57–0.95) (data not shown in the table). Furthermore, older age at first birth (>32 years) compared with younger age (<23 years) was significantly associated with reduced premenopausal risk of fibroadenoma (HR, 0.59; 95% CI, 0.40–0.87), and having breastfed for less than 3 months, compared with more than 12 months, was significantly associated with a reduced risk of FCCs in perimenopausal women (HR, 0.71; 95% CI, 0.52–0.96).

#### Body Mass Index

Overall, obesity (BMI >30) was associated with reduced risk of all BBDs, except EP (HR, 0.74; 95% CI, 0.55-1.00) (Table 1; eFigure in the Supplement). At premenopausal ages, being obese was associated with a reduction in the risk of EPA of 69% (HR, 0.31; 95% CI, 0.17–0.56) and by 44% for fibroadenoma (HR, 0.56; 95% CI, 0.41–0.78). For FCCs, the associated risk reductions were observed at both premenopausal (HR, 0.53; 95% CI, 0.43–0.65) and perimenopausal (HR, 0.48; 95% CI, 0.35–0.65) ages and in all age groups for cysts (premenopausal: HR, 0.53; 95% CI, 0.40–0.71; perimenopausal: HR, 0.43; 95% CI, 0.33–0.56; and postmenopausal: HR, 0.52; 95% CI, 0.30–0.89).

#### Oral Contraceptives

The risk of fibroadenoma at premenopausal ages was significantly associated with current (HR, 0.65; 95% CI, 0.47–0.90) or previous (HR, 0.75; 95% CI, 0.58–0.97) use of oral contraceptives for 8 or more years (Table 2). Furthermore, in premenopausal women, previous use of oral contraceptives of less than 8 years (HR, 1.32; 95% CI, 1.00–1.72) was associated with a significant reduction in the risk of EP, but no other significant associations were observed (Table 2).

**Table 2.** Risk of Benign Breast Diseases by the Use of Oral Contraceptives and HRT

Hormonal factor by age group^a^	HR (95% CI)^b^				
	Epithelial proliferation		Fibroadenoma	Fibrocystic changes	Cyst
	With atypia	Without atypia			
Ever use of oral contraceptives^c^					
Premenopausal	1.00 (0.73-1.36)	1.15 (0.90-1.46)	0.83 (0.67-1.03)	1.03 (0.89-1.19)	1.03 (0.84-1.27)
Perimenopausal	0.86 (0.64-1.16)	1.18 (0.89-1.58)	1.20 (0.87-1.65)	0.98 (0.81-1.19)	0.98 (0.83-1.16)
Previous use of oral contraceptives^c^					
Premenopausal	1.03 (0.74-1.42)	1.24 (0.97-1.59)	0.83 (0.67-1.04)	1.08 (0.93-1.25)	1.09 (0.88-1.35)
Perimenopausal	0.87 (0.65-1.17)	1.20 (0.90-1.60)	1.23 (0.89-1.70)	0.98 (0.80-1.19)	0.99 (0.84-1.16)
Previous use for <8 y					
Premenopausal	0.97 (0.66-1.41)	1.32 (1.00-1.72)^d^	0.90 (0.70-1.16)	1.13 (0.95-1.33)	1.17 (0.92-1.47)
Perimenopausal	0.95 (0.67-1.34)	1.37 (0.99-1.89)	1.35 (0.94-1.93)	1.01 (0.81-1.27)	1.06 (0.88-1.28)
Previous use for ≥8 y					
Premenopausal	1.08 (0.76-1.54)	1.16 (0.88-1.54)	0.75 (0.58-0.97)^d^	1.04 (0.88-1.22)	1.02 (0.80-1.29)
Perimenopausal	0.82 (0.59-1.13)	1.07 (0.79-1.47)	1.16 (0.82-1.64)	0.95 (0.77-1.18)	0.94 (0.79-1.12)
Current use					
Premenopausal	0.91 (0.60-1.38)	0.93 (0.69-1.27)	0.83 (0.63-1.09)	0.92 (0.77-1.11)	0.85 (0.64-1.12)
Perimenopausal	0.67 (0.32-1.40)	0.96 (0.49-1.87)	0.59 (0.25-1.38)	1.05 (0.70-1.58)	0.89 (0.61-1.31)
Current use for <8 y					
Premenopausal	0.71 (0.37-1.36)	0.99 (0.65-1.52)	1.24 (0.86-1.79)	1.01 (0.77-1.31)	0.93 (0.61-1.40)
Perimenopausal	1.38 (0.43-4.42)	1.53 (0.48-4.89)	1.61 (0.50-5.17)	0.80 (0.30-2.17)	1.09 (0.52-2.33)
Current use for ≥8 y					
Premenopausal	0.99 (0.64-1.55)	0.91 (0.64-1.28)	0.65 (0.47-0.90)^d^	0.89 (0.73-1.09)	0.82 (0.60-1.12)
Perimenopausal	0.51 (0.20-1.27)	0.82 (0.38-1.81)	0.36 (0.11-1.16)	1.11 (0.72-1.72)	0.85 (0.55-1.30)
Use of HRT in postmenopausal women^c^					
Ever use	1.81 (1.07-3.07)^d^	1.68 (0.93-3.04)	1.21 (0.51-2.88)	1.60 (1.03-2.48)^d^	1.98 (1.40-2.81)^d^
Previous use	1.46 (0.63-3.37)	1.60 (0.64-3.98)	0.50 (0.06-3.97)	0.68 (0.25-1.88)	1.47 (0.77-2.78)
Previous use for <5 y	1.70 (0.54-5.35)	1.43 (0.35-5.79)	1.17 (0.15-9.13)	0.78 (0.19-3.15)	1.21 (0.44-3.32)
Previous use for ≥5 y	1.30 (0.41-4.15)	1.74 (0.54-5.61)	NA^e^	0.59 (0.14-2.46)	1.66 (0.75-3.66)
Current use	2.00 (1.10-3.64)^d^	1.72 (0.87-3.42)	1.58 (0.63-4.00)	2.02 (1.27-3.22)^d^	2.20 (1.51-3.21)^d^
Current use for <5 y	1.51 (0.54-4.19)	1.88 (0.67-5.27)	NA^e^	2.59 (1.38-4.86)^d^	2.40 (1.38-4.17)^d^
Current use for ≥5 y	2.26 (1.15-4.46)^d^	1.65 (0.73-3.72)	2.61 (1.02-6.69)^d^	1.68 (0.92-3.06)	2.09 (1.33-3.28)^d^

Abbreviations: HR, hazard ratio; HRT, hormone replacement therapy; NA, not applicable.

^a^ Premenopausal ages are 25 to 44 years; perimenopausal ages, 45 to 54 years; and postmenopausal ages, 55 to 69 years.

^b^ Cox proportional hazards regression with time-varying covariates using age as the time scale adjusted for Karolinska Mammography Project for Risk Prediction of Breast Cancer unit, birth cohort, and educational level.

^c^ Current use includes current use and up to 4 years since last use, and previous use includes 5 years or more since last use. Never users were used as the reference group.

^d^ Significant association at *P* < .05.

^e^ Too few cases in this category to be analyzed in fibroadenoma.

#### Hormone Replacement Therapy

Postmenopausal ever HRT use was significantly associated with an increased risk of EPA (HR, 1.81; 95% CI, 1.07–3.07), FCCs (HR, 1.60; 95% CI, 1.03–2.48), and cysts (HR, 1.98; 95% CI, 1.40–2.81) (Table 2). That association was restricted to current HRT users for cysts (HR, 2.20; 95% CI, 1.51–3.21), independent of duration of use, whereas for EPA, the association was strongest in current users with 5 or more years of use (HR, 2.26; 95% CI, 1.15–4.46) and for FCC with less than 5 years of use (HR, 2.59; 95% CI, 1.38–4.86). Furthermore, current HRT use of 5 or more years was significantly associated with increased risk of fibroadenoma (HR, 2.61; 95% CI, 1.02–6.69).

### Risk of BBDs by Family History of Breast Cancer

Family history of breast cancer was significantly associated with increased risk of BBDs (Table 3).

**Table 3.** Risk of Benign Breast Diseases by Family History of Breast Cancer in Mother or Sister

Age group^a^	HR (95% CI)^b^				
	Epithelial proliferation		Fibroadenoma	Fibrocystic changes	Cyst
	With atypia	Without atypia			
Premenopausal	2.11 (1.48-3.00)^c^	1.90 (1.46-2.49)^c^	1.51 (1.13-2.00)^c^	1.39 (1.14-1.69)^c^	1.41 (1.09-1.83)^c^
Perimenopausal	1.43 (0.99-2.06)	1.68 (1.24-2.28)^c^	1.97 (1.44-2.70)^c^	1.26 (0.99-1.62)	1.42 (1.17-1.72)^c^
Postmenopausal	1.13 (0.58-2.21)	1.54 (0.80-2.96)	2.55 (1.20-5.42)^c^	1.30 (0.78-2.16)	1.14 (0.72-1.79)

Abbreviation: HR, hazard ratio.

^a^ Premenopausal ages are 25 to 44 years; perimenopausal ages, 45 to 54 years; and postmenopausal ages, 55 to 69 years.

^b^ Cox proportional hazards regression with time-varying covariates using age as the time scale adjusted for Karolinska Mammography Project for Risk Prediction of Breast Cancer unit, birth cohort, educational level, and number of sisters.

^c^ Significant association at *P* < .05.

At premenopausal ages, family history of breast cancer was associated with double the risk of EPA (HR, 2.11; 95% CI, 1.48-3.00) and increased risk of FCC (HR, 1.39; 95% CI, 1.14-1.69) and EP (HR, 1.90; 95% CI, 1.46-2.49). The risk of EP was also increased at perimenopausal ages (HR, 1.68; 95% CI, 1.24-2.28). At both premenopausal and perimenopausal ages, family history of breast cancer was associated with an approximately 40% increased risk of cysts (premenopausal: HR, 1.41; 95% CI, 1.09-1.83; perimenopausal: HR, 1.42, 95% CI, 1.17-1.72). Furthermore, family history of breast cancer was associated with the risk of fibroadenoma in all age-groups (premenopausal: HR, 1.51; 95% CI, 1.13-2.00; perimenopausal: HR, 1.97; 95% CI, 1.44-2.70; postmenopausal: HR, 2.55; 95% CI, 1.20–5.42).

## Discussion

This cohort study of 61 617 women presents up-to-date incidence rates for BBDs and found that the associated risks of the most common BBDs cover a wide and relatively young age range. Furthermore, the study found that hormonal factors, well known to be associated with breast cancer risk, were associated with the risk of BBDs, as was family history of breast cancer, and that, as hypothesized, the risk was influenced by age.

It is difficult to directly compare the incidence rates in this study with those in older studies. For instance, several distinct benign conditions were often combined into the less well-defined diagnosis of fibrocystic breast diseases.^1^ Moreover, reproductive and lifestyle factors have changed over time. The mean age at first birth is considerably higher today, and as was observed in this study, parity-related factors, such as number of births and age at first birth, were associated with the risk of BBDs. Mammographic screening enables more frequent detection of BBDs, and a possible interpretation is that the incidence rates reported in this study are higher than what older available studies have found.^3,4,5^ However, this study’s estimates are lower than the estimation from autopsy studies suggesting that every second woman will have a BBD,^1,2^ which indicates that some BBDs may not be diagnosed, such as benign lesions that do not appear as well-defined solid tumors.

These findings suggest a heterogenous BBD risk pattern by the traditional hormonal breast cancer risk factors and that the risk of BBDs varies among the analyzed age groups. As observed in this and previous studies on BBDs^1,2^ and breast cancer,^12^ obesity generally is associated with a reduced premenopausal risk, with the exception of EP. Of interest, the risk reduction extended to older age groups for FCCs and cysts in this study, which may suggest that certain cysts generally are more difficult to detect in breasts with a larger amount of fat tissue. However, menstrual cycle patterns and BMI were reported only at the time of recruitment. Furthermore, this study and others^1,2^ have not found any association between age at menarche and the risk of BBDs, and irregular menstrual cycles have been found to be both more and less common in women with BBDs.^31,32^ In this study, having regular menstrual cycles was associated with an increased premenopausal risk of fibroadenoma, and at postmenopausal ages, a history of regular cycles was associated with an increased risk of EPA but a reduced risk of fibroadenoma. Furthermore, consistent with the findings in this study, oral contraceptives are generally protective against BBDs, especially with longer duration of use, whereas HRT increases the risk.^1,2^ It is, however, debated whether the protective effect and increase in risk extend to atypical disease and how different doses affect the risk.^33,34,35,36,37^ This study found a protective association between fibroadenoma and long use of oral contraceptives (≥8 years) at premenopausal ages, whereas HRT was associated with an increased postmenopausal risk of EPA, fibroadenoma, FCCs, and cysts. Thus, additional studies are needed to determine how exogenous hormone use affects the risk of BBDs.

A finding inconsistent with previous studies^1,2^ was the associated reduced risk of EP in nulliparous premenopausal women, which suggests that being pregnant is, in the short term, associated with an increased risk of nonatypical proliferative benign disease. In contrast, previous studies^1,2^ have mainly observed higher risk of BBDs in nulliparous women, similar to breast cancer. In addition, previous studies^1,2,3^ have generally not found any associations with age at first birth or breastfeeding duration, which in this study were associated with the risk of fibroadenoma at premenopausal ages and FCCs at perimenopausal ages. Notably, in premenopausal women, being nulliparous was associated with an increased risk of cysts when compared with parity of 3 or more but a reduced risk when compared with being uniparous, which might indicate that several full-term pregnancies are needed to reach a protective effect against developing cysts.

Furthermore, this study found that family history of breast cancer, a strong breast cancer risk factor, was associated with proliferative and nonproliferative diseases, predominantly at premenopausal ages. Proliferative benign diseases are associated with an increased risk of breast cancer; however, risk estimates vary among studies,^7,9,16,17^ and it is not clear whether nonproliferative diseases impact the risk. To identify individuals susceptible to breast changes, it would be helpful to ascertain which women with a BBD diagnosis are at elevated risk of breast cancer and who is not, which could include information on the woman’s history of hormonal factors.

### Limitations

This study has limitations. Smaller studies are able to have more detailed, prospectively assessed pathological data, although such data come with lower statistical power and poorer disease classification. Use of the KARMA study and the Swedish personal identification numbers allowed the unique combination of quality-registry data, questionnaire data, and pathology medical records on breast biopsies, which made it possible to analyze a large number of biopsy-confirmed BBDs in a large number of women and to follow up the participants for a long time. The BBD diagnoses obtained from SymPathy comprise biopsies taken on referral after mammographic screening as well as after clinical examination, but information on mode of detection is not available. However, most BBD diagnoses in patients between the ages of 40 and 69 years will be from mammographic screening. As with all survey studies, self-reported retrospective questionnaire data are of varying quality; however, the educational level in Sweden is high. The KARMA cohort is population based because all women attending mammographic screening were invited; however, even though mammographic screening attendance in Sweden is high, the women needed to have actively chosen to participate in the study. Women with higher educational level or family history of breast cancer are more likely to accept the invitation and attend mammographic screening and to agree to participate in studies. Furthermore, the large number of statistical tests performed in this study generates a risk of false-positive results by random chance; thus, the findings need to be replicated in other large cohort studies.

## Conclusions

This cohort study estimated the incidences of BBDs and found that the risk of BBDs is associated with hormonal factors and age. Interestingly, the risks of proliferative and nonproliferative BBDs were associated with family history of breast cancer but not with all traditionally recognized hormonal breast cancer risk factors. The results for hormonal risk factors are to some extent consistent with previous studies^1,2^ but also highlight new findings that need further investigation. Indeed, the etiology of BBDs is a subject that has been relatively unexplored during the last decades. Further work on BBDs with updated classification is needed to determine risk factors for BBDs and to understand the differences and associations between benign and malignant breast diseases.

## Notes

Supplement. **eMethods.** Supplemental Methods **eTable 1.** Classification of Benign Breast Diseases (BBDs) **eTable 2.** Baseline Characteristics at Time of Recruitment of the KARMA Participants Included in the Study **eTable 3.** Age Incidence Rates per 100 000 Person-Years in KARMA **eFigure.** Risk of Benign Breast Diseases (BBDs) by Hormonal Factors
