# Supplementary material for: Characterization of Benign Breast Diseases and Association With Age, Hormonal Factors, and Family History of Breast Cancer Among Women in Sweden
Source: JAMA Netw Open. 2021 Jun 25;4(6):e2114716. doi: 10.1001/jamanetworkopen.2021.14716 (PMC8233703; doi:10.1001/jamanetworkopen.2021.14716)
Supplement: Supplement. — eMethods. Supplemental Methods eTable 1. Classification of Benign Breast Diseases (BBDs) eTable 2. Baseline Characteristics at Time of Recruitment of the KARMA Participants Included in the Study eTable 3. Age Incidence Rates per 100 000 Person-Years in KARMA eFigure. Risk of Benign Breast Diseases (BBDs) by Hormonal Factors [file jamanetwopen-e2114716-s001.pdf]

Supplementary Online Content

Johansson A, Christakou AE, Iftimi A, et al. Characterization of benign breast diseases and association with age, hormonal factors, and family history of breast cancer among women in Sweden. *JAMA Netw Open*. 2021;4(6):e2114716. doi:10.1001/jamanetworkopen.2021.14716

eMethods. Supplemental Methods

eTable 1. Classification of Benign Breast Diseases (BBDs)

eTable 2. Baseline Characteristics at Time of Recruitment of the KARMA Participants Included in the Study

eTable 3. Age Incidence Rates per 100 000 Person-Years in KARMA

eFigure. Risk of Benign Breast Diseases (BBDs) by Hormonal Factors

This supplementary material has been provided by the authors to give readers additional information about their work.

© 2021 Johansson A et al. *JAMA Network Open.*

eMethods. Supplemental Methods

Classification of benign breast diseases (BBDs)

The pathology medical records on breast biopsies in SymPathy include a diagnosis code (morphology codes from the International Classification of Diseases for Oncology, ICD-O[1], system and the Systematized Nomenclature of Medicine, SNOMED[2]), and describing diagnosis text. From the more specific diagnoses in SymPathy, following ten BBD subtypes were included in the study: epithelial proliferation with atypia (EPA, i.e. atypical hyperplasia or dysplasia), epithelial proliferation without atypia (EP, i.e. hyperplasia), adenosis (including sclerosing adenosis), papilloma (single and multiple, mainly specified as intraductal), breast calcifications, fibroadenoma (including giant fibroadenoma, hamartoma, tubular adenoma, and adenoma of the nipple), cysts, fibrocystic changes (FCC; except 'fibrocystic changes', the most common included diagnoses were 'fibrosis' and 'cystic fibroadenosis'), inflammation (chronic and granulomatous), and non-epithelial tumors (with lipoma as the most common diagnosis). The classification of the BBD subtypes are according to the latest European guidelines[3] and was done in consultation with an experienced breast pathologist (L.S). For more details, see **eTable 1**.

Breast biopsy records were excluded if being of normal morphology, no diagnosis (or if not enough material for diagnosis), unspecific (e.g. unspecified adenoma or benign tumor), not breast cells (e.g. squamous cells), or likely to have been caused by external factors (e.g. acute inflammation, bleeding, foreign body reaction). Furthermore, breast carcinoma *in situ* (BCIS) and all malignant biopsies or with any suspicion on malignancy (including Paget's disease) were excluded. If a participant had been diagnosed with the same BBD subtype multiple times, we only considered the first diagnosis. Furthermore, all BBDs diagnosed the same year or after any breast cancer diagnosis were excluded, leaving 7,067 diagnoses for 5,341 unique participants.

Breast cancer diagnoses

Breast cancer diagnoses were obtained from the National Swedish Cancer Register (Swecan) by the use of the following ICD/ICD-O codes ICD-7 [170, 1701, 1702, 1707-1709], ICD-9 [1741-1744, 1749], ICD-10 [C500-C506, C508, C509] and ICD-O-3 [C500-C506, C508, C509].

Hormonal factors and covariates from the KARMA questionnaire

All covariates included in the survival analyses were obtained from the web-based questionnaire that the participants took part in upon recruitment. Participants were divided by birth cohort (<1950, 1950-1965, >1965) and BMI was calculated from self-reported height and weight at recruitment and categorized into underweight (BMI<18.5), normal weight (BMI 18.5-25), overweight (BMI >25-30) and obese (BMI>30.0).

<u>Educational level</u>

The participants were asked for their highest educational level, choosing between compulsory elementary school (nine-year 'grundskola'), the previous Swedish elementary school system 'folkskola' or 'realskola', upper secondary school (gymnasium), and university, or provide another written answer. All written answers (n=8,521) were reviewed and categorized into if the participant had attended university or not. Alternative post-secondary courses, including 'komvux' (municipal adult education), 'folkhögskola' and 'yrkeshögskola' (higher vocational education), were not considered university-level education. Instead, if the participant had provided their profession and if university education was required for the job (according to today's requirements) they were categorized as having attended university (e.g. nurse, midwife, teacher), and otherwise not (e.g. assistant nurse or secretary). If the written answer was unspecific (e.g. post-secondary education [eftergymnasial utbildning]) they were categorized as of unknown educational level.

<u>Age at menarche</u>

Age at menarche was extracted from the age the participants stated they had their first menstrual period, and categorized as <12, 12-14, and >14.

<u>Menstrual cycle regularity and length</u>

The participants were asked if they during their adult-life have had periods each month and if they were able to predict the first day of their period with an accuracy of 5 days, and categorized as having regular or irregular cycles. Further, participants with regular cycles were asked how many days they usually had in between their periods. The pre-defined options for cycle length were ≤22, 23-26, 27-30, 31-34, 35-38, and ≥39 days. In the study, cycle length was categorized as <27 days, 27-30 days, and >30 days.

<u>Parity, age at first birth and duration of breastfeeding</u>

For the parity-related factors, participants were firstly asked how many children they had given birth to and for each delivered child they were further asked for the birth year and time breastfeeding. Two or more identical

© 2021 Johansson A et al. *JAMA Network Open.*

birth years (e.g. twins or triplets) were added to the parity as multiple births, and only the maximum provided time breastfeeding was used. Parity was categorized as 0, 1, 2 or ≥3 children and age at first birth was categorized as <23, 23-32, and >32. The pre-defined options for breastfeeding duration in the questionnaire were 0, <1, 1-6, 7-12, or >12 months breastfeeding. These options were re-coded as 0, 0.5, 3.5, 9.5, and 12.1 months. The cumulative breastfeeding duration, i.e. the total sum of the breastfeeding time for all children, was calculated. For multiple births (e.g. twins or triplets) only the maximum provided time breastfeeding was used in the calculation. Breastfeeding duration was analyzed in the categories 0-3, 3-12, and >12 months.

Oral contraceptives

The assessment on oral hormonal contraceptives included combination pills (contains progestin and estrogen) and mini pills (contains only progestin). In the questionnaire, the participants could choose between following pre-defined contraceptives: combination pills, mini pills, injection, implant, hormonal, copper or other kind of IUD, or others, where they were asked to provide a written answer. All written answers (n=2,515) were reviewed and categorized into if they were oral contraceptives or not.[4-7] Ever use of oral contraceptives were determined from if the participant had selected combination or mini pills from the pre-defined list or provided an oral contraceptive in the written answer.

For each selected contraceptive, including the written answer, the participants were further asked to provide age at start and duration of use. The pre-defined options in the questionnaire for duration were 0-3 months, 3-24 months, 2-5 years, 6-10 years, 11-15 years, 16-20 years, and more than 20 years. The options were re-coded as 0, 1, 3.5, 8, 13, 18, and 21 years. For each oral contraceptive, the start age and stop age (calculated as duration added to the start age) were extracted. Oral contraceptive use was analyzed by status of use (never use, ever use, current use, previous use) and duration. Current use included up to 4 years since stop and previous use was defined as 5 or more years since stop. The duration was split by 8 years, since this was the median duration use of oral contraceptives in the KARMA cohort.

Hormone replacement therapy (HRT)

The use of HRT in the form of oral tablets was assessed in this study. Participants were asked if they ever had used hormones for another purpose than for contraceptive use, and divided into if they had used hormones for the menopause (defined by either a clearly defined answer due to menopause or removal of both ovaries) or unknown reason. Participants that only had used hormones for other reasons than the menopause were set as not taken HRT. Participants were further asked to provide what kind of hormonal treatment they had taken, choosing between tablets, local treatments, patch, and injections. Participants that selected hormonal tablets were further asked to indicate which tablets they had used from a pre-defined list of 25 different HRT tablets or to provide a written answer. All written answers (n=1,914) were reviewed.

Participants that had used hormones for the menopause and that had selected an HRT tablet or provided and HRT tablet in their written answer were classified as having used HRT. If the reason for the hormonal treatment was less clear, the participants were classified after review of which tablet(s) they had selected or provided in writing (this was done in consultation with an experienced gynecologist K.R-W.). If they only had selected tablets that might be used for other reason than menopause (for instance, progestin-only tablets can also be used to regulate heavy bleeding, treatment of endometriosis, or in infertility treatment, and estradiol-only tablets can be used in frozen embryo transfer[4,6,8-10]) the HRT usage was set as unknown. If they on the other hand had selected tablet(s) that are mainly/only used for HRT, they were classified as taken HRT. The HRT use for participants that did not specify which tablet(s) they had used was set as unknown, since many non-hormonal tablets can be used for menopausal problems.

Furthermore, participants were asked to provide which year they started and stopped taking HRT and the total duration. The pre-defined options for the duration was ‘less than 1 year’, followed by 1-year intervals between 1-25 years, as well as >25 years. The ‘<1 year’-option was re-coded as 0 years (since this can mean anything between a very short period to 12 months). Similar to oral contraceptives, HRT was analyzed by the status of use (never use, ever use, current use up to 4 years since stop, previous use ≥5 years since stop) and duration. The duration was split by 5 years, since it was more common to use HRT for a few years.

Family history of breast cancer

The participants were asked whether any of their biological parent or siblings had been diagnosed with breast cancer, and to estimate at which age they were diagnosed. From this, the information on whether the participant’s mother or any full sister had been diagnosed with breast cancer was extracted. If age at diagnosis was missing for the mother or sister it was set as 65.

© 2021 Johansson A et al. *JAMA Network Open.*

Time-varying covariates

The risk of BBDs by hormonal factors and family history of breast cancer was assessed by Cox proportional hazard regression with time-varying covariates,[11] using R function 'tmerge' from package 'survival'. For instance, parity was allowed to change over time (age) by the participants age at birth for each child. Before a participants age at first birth, she was categorized as nulliparous, which was changed to a parity of 1 when she gave birth to her first child, which was changed to a parity of 2 when she gave birth to her second child, etc. Total cumulative breastfeeding duration was allowed to change over time in a similar way, by the use of the participants' age at birth and duration of breastfeeding for each child. For oral contraceptives and HRT, the status of use (never use, ever use, current use, previous use) and duration were allowed to change by the information on age at start, duration, and age at stop. Furthermore, family history of breast cancer was allowed to change at the age when the participant's mother or sister had been diagnosed with breast cancer. Given that we don't have the information on age at birth for the participants' mothers, one generation was approximated to 30 years, i.e. the mother was considered as being 30 years older than the participant. Sisters were approximated to the same age as the participant, assuming a similar probability of younger and older sisters.

Supplemental References

1. World Health Organization. *International Classification of Diseases for Oncology (ICD-O)*. Third ed. 2013.
2. SNOMED International. Leading heatlhcare terminology, worldwide. Accessed April 2020, https://www.snomed.org
3. Writing Group for NHS Breast Screening Programme and the Royal College of Pathologists. *Pathology reporting of breast disease in surgical excision specimens incorporating the dataset for histological reporting of breast cancer*. 2016.
4. Bergendal A. *Venous thromboembolism in women : an assessment of hormonal, genetic and other risk factors*. Karolinska Institutet; 2013. https://openarchive.ki.se/xmlui/handle/10616/41511
5. Dumeaux V, Alsaker E, Lund E. Breast cancer and specific types of oral contraceptives: a large Norwegian cohort study. *International journal of cancer*. Jul 20 2003;105(6):844-50. doi:10.1002/ijc.11167
6. FASS. Accessed March 2020, https://www.fass.se/LIF/startpage
7. IARC Working Group on the Evaluation of Carcinogenic Risks to Humans. Combined estrogen-progestogen contraceptives and combined estrogen-progestogen menopausal therapy. *IARC monographs on the evaluation of carcinogenic risks to humans*. 2007;91:1-528.
8. Behandling med HRT. Behandlingsrekommendationer (Treatment with HRT. Therapeutic recommendations). *Information från Läkemedelsverket*. 2004;15(3)
9. Persson I, Adami HO, Lindberg BS, Johansson ED, Manell P. Practice and patterns of estrogen treatment in climacteric women in a Swedish population. A descriptive epidemiological study. Part I. *Acta obstetricia et gynecologica Scandinavica*. 1983;62(4):289-96. doi:10.3109/00016348309156224
10. The Swedish Council on Technology Assessment in Health Care. Behandling med östrogen (Treatment with estrogen). 2002;
11. Zhang Z, Reinikainen J, Adeleke KA, Pieterse ME, Groothuis-Oudshoorn CGM. Time-varying covariates and coefficients in Cox regression models. *Annals of translational medicine*. Apr 2018;6(7):121. doi:10.21037/atm.2018.02.12

© 2021 Johansson A et al. *JAMA Network Open.*

eTable 1. **Classification of benign breast diseases (BBDs).** The BBD diagnoses of breast biopsies for the KARMA obtained from the pathology medical record system SymPathy.

**BBD subtype**	**Diagnosis codes**	**Diagnosis text**
Epithelial proliferation with atypia	M49061, M49251	Radial scar
	M697, M69700, M69701, M69030	Atypia
	M69706	Atypia light
	M69707	Atypia moderate
	M69708	Atypia strong
	M6972, M69720	Atypical columnar cell change
	M69726	Atypical columnar cell change light
	M69727	Atypical columnar cell change moderate
	M69728	Atypical columnar cell change strong
	M6978, M69780	Inflammatory atypia
	M72005	Atypical hyperplasia
	M72105	Atypical hyperplasia lobular
	M72175	Atypical hyperplasia ductal
	M72425, M74245	Atypical hyperplasia adenomatous
	M74, M74000	Dysplasia
	M74006	Dysplasia light
	M74007	Dysplasia moderate
	M74008	Dysplasia strong
	M74300	Dysplasia cystic
	M74325	Atypical cystic fibroadenosis
	M76085	Atypical epithelial proliferation
Epithelial proliferation without atypia	M72, M72000	Hyperplasia
	M721, M72100	Hyperplasia lobular
	M72170	Hyperplasia intraductal
	M722, M72200	Hyperplasia lymphoid
	M7242	Adenomatous hyperplasia
	M7248	Hyperplasia microglandular
	M76, M76000	Proliferation
	M7608, M76080	Epithelial proliferation
	M85000	Ductal epithelial proliferation benign
Adenosis	M74200	Adenosis
	M7422, M74220	Adenosis sclerosing
	M74240	Adenosis blunt duct
	M7426, M74260	Adenosis florid
Papilloma	M80500	Papilloma
	M82600	Papillary adenoma
	M8503, M85030	Intraductal papilloma
	M85031	Suspected intraductal papilloma
	M85050	Papillomatos / Intraductal Papillomatos
	M89830	Adenomyoepiteliom
Calcifications	M554, M55400	Calcifications
Fibroadenoma	M755, M75500	Hamartoma
	M82100	Adenoma tubular
	M85060	Adenoma of the nipple
	M90100	Fibroadenoma

© 2021 Johansson A et al. *JAMA Network Open.*

	M90200	Giant fibroadenoma
Fibrocystic changes	M321, M32100	Duct ectasia
	M332, M33200	Mucocele
	M3322, M33220	Galactocoele
	M49, M49000, M49001	Fibrosis
	M73, M73000	Metaplasia
	M7305, M73050	Metaplasia oxyphil cells / oncocytic
	M73310	Apocrine metaplasia
	M7432, M74320	Fibrocystic changes
	M743, M74300	Cystic fibroadenosis
	M761, M76100	Fibromatosis
Cyst	M4717, M47170	Lymphocytic infiltration of cells
	M33, M33000, M33300	Retention
	M334, M33400	Cyst
	M33420	Epithelial cyst
Inflammation	M421, M42100	Inflammation acute and chronic
	M43, M43000	Inflammation chronic
	M44, M44000	Inflammation granulomatous
	M44110	Reparative giant cells granuloma
	M44200	Inflammation granulomatous not necrotic
	M47000	Infiltration of inflammatory cells
Non-epithelial tumors	M4444	Granuloma telangiect
	M88100	Fibroma
	M88300	Fibrous histiocytoma
	M88320	Dermatofibroma
	M88500	Lipoma
	M88510	Fibrolipoma
	M88800	Hibernoma
	M88900	Leiomyoma
	M91200	Hemangioma
	M91210	Hemangioma cavernous
	M95400	Neurofibroma
	M95600	Neurilemmoma

© 2021 Johansson A et al. *JAMA Network Open.*

eTable 2. Baseline characteristics at time of recruitment of the KARMA participants included in the study.

**Baseline characteristics**	**KARMA study (n=61,617)**
**Age at baseline**, mean (sd)	53.8 (8.9)
**Birth cohort**, No (%)	
< 1950	14338 (23.3)
1950 - 1965	29034 (47.1)
> 1965	18245 (29.6)
Missing	0 (-)
**KARMA unit**, No (%)	
Södersjukhuset	31452 (51.0)
Helsingborg	16784 (27.2)
Lund	8298 (13.5)
Landskrona	5083 (8.2)
Missing	0 (-)
**Educational level**, No (%)	
University	32209 (54.5)
Elementary/Intermediate	26846 (45.5)
Unknown	2562 (-)
**Age at menarche**, No (%)	
< 12	7996 (13.3)
12 - 14	42772 (71.4)
> 14	9143 (15.3)
Missing	1706 (-)
**Menstrual cycle patterns**, No (%)	
Irregular cycles	7178 (12.8)
Regular cycles	48879 (87.2)
Missing	5560 (-)
Cycle length	
< 27 days	11753 (24.0)
27 - 30 days	32140 (65.8)
> 30 days	4986 (10.2)
**Parity,** No (%)	
Nulliparous	7821 (12.8)
Parous	53194 (87.2)
Missing	602 (-)
Number of children	
1 child	9037 (17.0)
2 children	29177 (54.9)
≥3 children	14980 (28.2)
**Age at first birth** [a], No (%)	
< 23	10601 (19.9)
23 - 32	33703 (63.4)
> 32	8890 (16.7)
Missing	602 (-)
**Breastfeeding duration** [a], No (%)	
< 3 months	4369 (8.2)
3 - 12 months	21774 (41.1)
> 12 months	26870 (50.7)
Missing	783 (-)
**BMI,** No (%)	

© 2021 Johansson A et al. *JAMA Network Open.*

Underweight (< 18.5 kg/m^2)	610 (1.0)
Normal (18.5 - 25.0 kg/m^2)	33508 (55.0)
Overweight (25.1 - 30.0 kg/m^2)	19006 (31.2)
Obese (> 30 kg/m^2)	7774 (12.8)
Missing	719 (-)
**Oral contraceptives**, No (%)	
No	10942 (18.0)
Yes	49926 (82.0)
Missing	749 (-)
Duration	
1 - 5 years	16544 (35.1)
6 - 10 years	16440 (34.9)
> 10 years	14148 (30.0)
Unknown	2794 (-)
Type	
Estrogen + progestin	44725 (89.8)
Progestin	5069 (10.2)
Unknown	132 (-)
**HRT**, No (%)	
No	50989 (88.4)
Yes	6696 (11.6)
Missing	3932 (-)
Duration	
1 - 5 years	2513 (45.8)
6 - 10 years	1673 (30.5)
> 10 years	1304 (23.8)
Unknown	1206 (-)
Type	
Estrogen + progestin	4475 (66.8)
Estrogen only	1394 (20.8)
Progestin only	359 (5.4)
Tibolone only	468 (7.0)
Unknown	0 (-)
**Family history of breast cancer**, No (%)	
Yes	7604 (12.9)
No	51524 (87.1)
Missing	2489 (-)

[a] Age at first birth and breastfeeding duration only for parous women

© 2021 Johansson A et al. *JAMA Network Open.*

eTable 3A. Age incidence rates per 100 000 person-years in KARMA.

	Epithelial proliferation with atypia		Epithelial proliferation without atypia		Fibroadenoma		Fibrocystic changes		Cyst	
**Age**	**Cases**	**Incidence rates**	**Cases**	**Incidence rates**	**Cases**	**Incidence rates**	**Cases**	**Incidence rates**	**Cases**	**Incidence rates**
**25**	6	15	13	32	18	45	17	42	3	7
**26**	7	17	14	33	11	26	23	55	3	7
**27**	2	5	15	34	9	21	14	32	8	18
**28**	4	9	8	18	12	26	35	77	9	20
**29**	3	6	12	25	14	30	24	51	20	42
**30**	2	4	14	28	20	41	16	33	20	41
**31**	8	16	23	45	18	35	34	66	12	23
**32**	6	11	14	26	15	28	49	92	15	28
**33**	6	11	20	36	20	36	59	107	28	51
**34**	13	23	13	23	24	42	67	117	19	33
**35**	10	17	25	42	27	46	74	125	23	39
**36**	7	12	39	64	30	49	60	99	27	44
**37**	12	19	28	45	28	45	80	130	31	50
**38**	18	29	34	55	34	55	77	125	32	52
**39**	16	26	39	63	27	44	80	130	38	62
**40**	23	37	34	55	50	81	86	140	62	101
**41**	23	37	29	47	48	78	94	153	48	78
**42**	32	52	50	81	52	85	112	182	68	111
**43**	28	46	51	84	33	54	115	188	86	141
**44**	36	61	35	59	45	76	109	185	98	167
**45**	43	76	36	64	45	80	101	179	97	172
**46**	32	59	49	90	53	98	97	179	110	202
**47**	33	63	36	69	41	79	91	175	107	205

© 2021 Johansson A et al. *JAMA Network Open.*

**48**	24	48	34	68	33	66	71	142	108	216
**49**	25	53	28	59	26	55	64	135	95	200
**50**	22	49	35	78	30	67	57	126	122	271
**51**	27	63	35	82	15	35	51	120	83	195
**52**	18	45	28	70	15	37	38	95	86	215
**53**	18	48	23	61	9	24	33	87	52	137
**54**	14	39	18	51	9	25	30	84	50	141
**55**	11	33	6	18	8	24	22	65	31	92
**56**	10	32	6	19	4	13	17	54	25	79
**57**	7	24	7	24	6	20	17	57	24	81
**58**	8	29	9	32	3	11	14	51	19	69
**59**	5	19	7	27	1	4	9	35	9	35
**60**	1	4	4	17	1	4	7	29	18	75
**61**	13	59	8	36	4	18	5	23	12	54
**62**	5	24	7	34	2	10	5	24	6	29
**63**	4	21	3	16	0	0	4	21	5	27
**64**	4	24	3	18	3	18	6	35	5	29
**65**	3	20	0	0	3	20	5	33	9	59
**66**	3	22	2	15	0	0	3	22	2	15
**67**	1	9	2	17	0	0	3	26	3	26
**68**	2	21	0	0	1	10	1	10	2	21
**69**	0	0	0	0	0	0	2	26	2	26
**Premenopausal ages, total nr of cases:**										
**25-44**	262		510		535		1225		650	
**Perimenopausal ages, total nr of cases:**										
**45-54**	256		322		276		633		910	
**Postmenopausal ages, total nr of cases:**										

© 2021 Johansson A et al. *JAMA Network Open.*

**55-69**	77	64	36	120	172

© 2021 Johansson A et al. *JAMA Network Open.*

eTable 3B. Age incidence rates per 100 000 person-years in KARMA.

	Adenosis		Papilloma		Calcification		Inflammation		Non-epithelial tumors	
**Age**	**Cases**	**Incidence rates**	**Cases**	**Incidence rates**	**Cases**	**Incidence rates**	**Cases**	**Incidence rates**	**Cases**	**Incidence rates**
**25**	1	2	0	0	0	0	0	0	0	0
**26**	4	10	0	0	0	0	0	0	0	0
**27**	3	7	0	0	0	0	2	5	1	2
**28**	2	4	1	2	0	0	2	4	2	4
**29**	1	2	0	0	0	0	2	4	0	0
**30**	7	14	0	0	1	2	2	4	1	2
**31**	7	14	0	0	0	0	1	2	2	4
**32**	5	9	3	6	0	0	1	2	1	2
**33**	11	20	1	2	0	0	0	0	1	2
**34**	3	5	0	0	0	0	3	5	3	5
**35**	6	10	3	5	0	0	3	5	4	7
**36**	7	12	3	5	0	0	3	5	4	7
**37**	9	15	6	10	0	0	2	3	1	2
**38**	9	15	2	3	0	0	0	0	3	5
**39**	7	11	1	2	0	0	1	2	3	5
**40**	12	20	4	7	2	3	2	3	2	3
**41**	12	20	6	10	3	5	3	5	6	10
**42**	16	26	5	8	1	2	4	7	6	10
**43**	16	26	8	13	0	0	7	11	4	7
**44**	13	22	8	14	4	7	3	5	3	5
**45**	18	32	10	18	3	5	4	7	0	0
**46**	11	20	10	18	1	2	2	4	7	13
**47**	8	15	13	25	2	4	2	4	6	12
**48**	9	18	13	26	3	6	0	0	4	8

© 2021 Johansson A et al. *JAMA Network Open.*

**49**	19	40	8	17	2	4	2	4	4	8
**50**	14	31	9	20	3	7	5	11	8	18
**51**	9	21	3	7	7	16	1	2	4	9
**52**	8	20	8	20	0	0	5	12	1	2
**53**	8	21	5	13	3	8	3	8	6	16
**54**	1	3	3	8	3	8	2	6	6	17
**55**	2	6	7	21	3	9	0	0	4	12
**56**	5	16	3	10	0	0	0	0	3	10
**57**	2	7	5	17	1	3	2	7	3	10
**58**	2	7	4	14	4	14	1	4	0	0
**59**	3	12	5	19	2	8	1	4	5	19
**60**	4	17	1	4	1	4	2	8	3	13
**61**	2	9	4	18	2	9	0	0	2	9
**62**	3	15	6	29	2	10	0	0	2	10
**63**	0	0	4	21	0	0	0	0	2	11
**64**	3	18	7	41	0	0	1	6	4	24
**65**	0	0	2	13	0	0	0	0	4	26
**66**	1	7	4	30	4	30	0	0	0	0
**67**	0	0	2	17	0	0	0	0	0	0
**68**	1	10	1	10	3	31	1	10	0	0
**69**	0	0	2	26	0	0	0	0	0	0
**Premenopausal ages, total nr of cases:**										
**25-44**	151		51		11		41		47	
**Perimenopausal ages, total nr of cases:**										
**45-54**	105		82		27		26		46	
**Postmenopausal ages, total nr of cases:**										
**55-69**	28		57		22		8		32	

© 2021 Johansson A et al. *JAMA Network Open.*

eFigure 1. Risk of benign breast diseases (BBDs) by hormonal factors. Multivariable Cox proportional hazard regression with time-varying covariates using age as the time-scale was used to estimate the risk of BBDs by hormonal factors, adjusting for KARMA unit, birth cohort, and educational level. The hazard ratios (HRs) and their 95% confidence intervals (CIs) are presented in forest plots for each BBD and hormonal factor. The analyses were divided in three age-groups, following women at pre- (25-44), peri- (45-54) and postmenopausal ages (55-69).

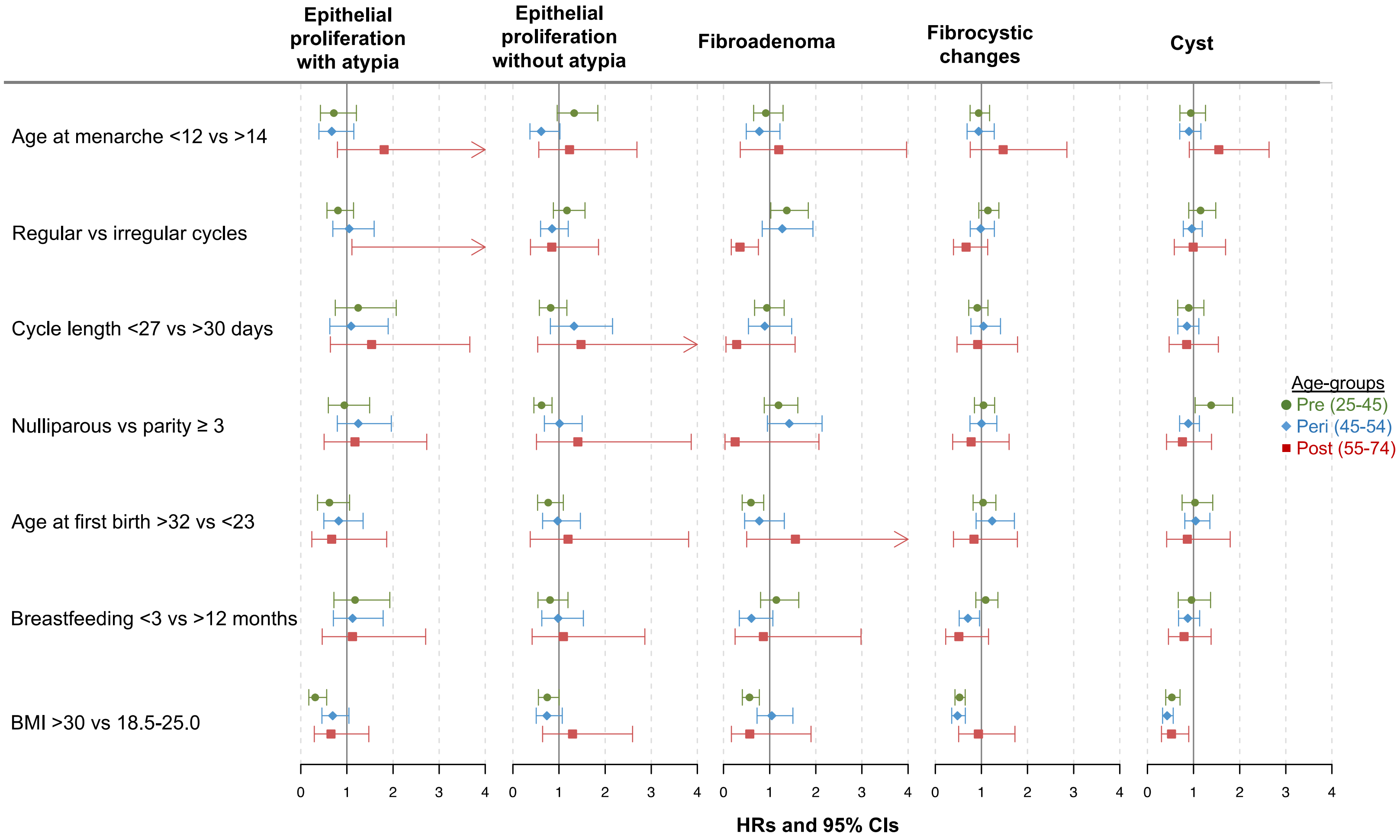

© 2021 Johansson A et al. *JAMA Network Open.*
